# Qualitative exploration of medical student experiences during the Covid-19 pandemic: implications for medical education

**DOI:** 10.1186/s12909-021-02726-4

**Published:** 2021-05-19

**Authors:** Helen Nolan, Katherine Owen

**Affiliations:** Warwick Medical School, Gibbet Hill, CV4 7AL Coventry, UK

**Keywords:** Community of practice, Social capital, Placement learning, Interprofessional learning

## Abstract

**Background:**

During the Covid-19 pandemic medical students were offered paid roles as medical student healthcare assistants. Anecdotal reports suggested that students found this experience rich for learning. Previous studies have explored alternative models of student service, however this defined medical student support role is novel.

**Methods:**

Individual semi-structured interviews were recorded with 20 medical students at a UK medical school exploring their experiences of placement learning and experiences of working as healthcare assistants. Responses were analysed qualitatively using a framework approach. The framework was developed into a model describing key findings and their relationships.

**Results:**

Interviews yielded data that broadly covered aspects of (1) Medical students’ experiences of clinical placement learning (2) Medical students’ experiences of working as medical student healthcare assistants (3) Learning resulting from working as a healthcare assistant (4) Hierarchies and professional barriers in the clinical environment (5) Influences on professional identity.

Participants described barriers and facilitators of clinical learning and how assuming a healthcare assistant role impacted on learning and socialisation within the multidisciplinary team. Students became increasingly socialised within the healthcare team, contributing directly to patient care; the resulting social capital opened new opportunities for learning, team working and enhanced students’ interprofessional identity. Students described the impact of these experiences on their aspirations for their future practice.

**Conclusions:**

Changes to work patterns in healthcare and delivery models of medical education have eroded opportunities for students to contribute to healthcare delivery and be embedded within a team. This is impacting negatively on student learning and socialisation and we suggest that medical curricula have much to learn from nursing and allied health professional training. Longitudinal embedment with a multidisciplinary team, where students have a defined role and work directly with patients may not only add value to clinical service, but also overcome current barriers to effective placement learning and interprofessional identity formation for medical students.

**Supplementary Information:**

The online version contains supplementary material available at 10.1186/s12909-021-02726-4.

## Background

As Covid-19 was classified as a global pandemic in March 2020, medical student clinical placements were paused to allow providers to focus exclusively on care delivery and to reduce transmission of infection [1]. Innovative online and technology-enhanced learning and assessments were rapidly trialled and implemented across the undergraduate medical education sector [2]. Telemedicine and teleteaching were implemented by many medical schools as mechanisms to provide some clinical learning opportunities [3], acknowledging that these cannot compensate for patient-student engagement opportunities, particularly for more junior students, whose clinical skills would still be in early development [4]. The need to continue medical training to provide a supply of junior doctors adequately experienced and equipped to provide safe medical care became an increasing concern.

Response and perspectives on the medical student role varied globally at this time [5–9]. Medical students have previously assumed greater responsibility and made valuable contributions during historic times of need, however in some areas medical students were viewed as non-essential in the healthcare environment [10]. Their potential role in the transmission of infection and the depletion of limited personal protective equipment (PPE) meant that they were excluded from clinical environments. However, with predictions of significant staff shortages as patient care needs increased and as health workers themselves became unwell, the possibility for medical students to support the clinical workforce was explored [2, 11]. Student response teams identified both clinical and non-clinical roles where medical students could assist and proposed this as a model that organisations could adopt, working with student volunteers [12]. Roles participating in patient care offered the greatest impact for both the overstretched health service and the developing professional. Such participation could continue to provide access to some authentic learning opportunities and help prevent clinical deskilling and deterioration in confidence. Careful consideration was required in identifying where students could effectively contribute without exceeding their competence levels.

A range of opportunities were accessed by students from our school, including a local paid volunteer scheme working as Medical Student Healthcare Assistants (MSHCAs), bank work in previous healthcare roles, remote triage and practical support (such as childcare) for NHS staff. Informal discussions with students suggested that the experience, though challenging, had been predominantly positive in terms of learning and socialisation within clinical teams, notably accessing opportunities not usually available to medical students. This study was therefore developed with the aim of understanding learning and personal or professional skill development achieved through MSHCA roles and barriers preventing students from participating. Definition of such outcomes may inform future education development.

## Methods

### Context

This study of medical student experiences of assuming a MSHCA role was part of a larger study on student placement learning experiences and barriers and facilitators to participation and was undertaken in one 4-year graduate-entry medical school in England. Students undertake some introductory clinical experiences in Year 1 and by Year 2 students transition to fulltime clinical placements [13]. A local scheme to enable 1st -3rd year students to undertake paid volunteering MSHCA roles was in place during the Covid-19 pandemic, similar to schemes in other areas. Early informal reports from MSHCAs (e.g., in tutor meetings and at student consultation events) suggested that students were accessing novel formative experiences, distinct from typical placement experiences. These appeared to be greatly valued by students, thus further exploration of their experiences and the formative impact was warranted. All students in year 2 & 3 were contacted via email, outlining the purpose of the study, and were invited to participate in an interview with either HN or KO. The study was granted approval by the University of Warwick Biomedical Sciences Research Ethics Committee.

### Study design and sampling

 Student volunteers were purposively sampled to ensure an equal mix of those choosing to participate in the scheme and those not, as we were interested in exploring barriers preventing students from taking part. Participant demographics are reported in Additional file 1. Informed consent was obtained from all the participants. Individual semi-structured interviews containing five open questions, with probing and exploration of responses to illicit full experience were undertaken, 10 interviews by each researcher. Interviews were conducted during July and August 2020, after the first wave of Covid-19 and by which time almost 300,000 Covid positive cases and over 40,000 Covid-related deaths had been recorded in the UK [14]. The interview schedule had been developed from anecdotal reports from students and review of existing literature (Additional file 2). After each researcher had completed two interviews the schedule and early findings were discussed, and iterative changes were made. Interviews were conducted and recorded on Microsoft Teams with video allowing nonverbal cues to be noted. Voice recognition software was used to produce an initial transcription which was then corrected by the researchers. Average interview duration was 32 min. Following this the recording was deleted. Transcripts were uploaded to NVivo12 (QSR International Pty Ltd, Doncaster, Australia). Following analysis participants were invited to comment on the results to minimise effects of potential researcher bias. No areas of dissonance were identified. All methods were performed in accordance with the relevant guidelines and regulations.

### Role of researchers

Both researchers developed the idea for the project, interviewed students and analysed data. KO has a directly student-facing leadership role within the medical programme and HN has a quality leadership role and is directly known to fewer students. There was an awareness throughout the interviews that our roles may impact students’ willingness to share experiences and students had the option to choose their interviewer, if desired, but this did not appear to be a concern. We were also aware of potential bias during the analysis and actively sought evidence of contrary views.

### Analysis

A constructivist approach was taken to data analysis as the aim was to understand how students decided to undertake a MSHCA role and how their individual experiences influenced this, how students understood their role as students and as MSHCAs, how they integrated into the healthcare setting and the impact of this experience. After 20 interviews, researchers noted consensus in preliminary themes, that no novel themes were emerging and agreed that theoretical saturation had been reached.

An inductive process using framework analysis [15, 16] was used to ensure a robust approach to data analysis and to minimise the impact of any researcher pre-conceptions. Both researchers familiarised themselves with the whole data set by reading all the interviews and independently coded 5 transcripts. Padlet, a secure online bulletin board, was used to facilitate a discussion of individual codes leading to formation of a framework. Each researcher then coded the whole data set against the framework using Nvivo 12. During analysis an additional framework code was added by mutual agreement. A second pass of the data was undertaken by both researchers to identify subthemes. Researchers both maintained reflective diaries during analysis which were discussed at weekly meetings. Interpretation of the data was also discussed and its relationship to existing theoretical frameworks. Evidence of data contradicting theoretical and iterative frameworks was actively sought.

The framework was developed into a model describing social & cognitive impact and exploring the relationships between themes.

## Results

Interviews yielded data that broadly covered aspects of (1) Medical students’ (baseline) experiences of clinical placement learning (2) Medical students’ experiences of working as MSHCAs (3) Learning resulting from working as MSHCAs (4) Hierarchies and professional barriers in the clinical environment and (5) Influences on professional identity. Data presented in themes 2,3, and 5 predominantly relates to the experiences of those undertaking MSHCA roles. Results relating to the other participant category are not presented in detail here. Quotes are presented according to participant number.

### Medical students’ prior experiences of clinical placement learning

Although students spoke of valuing and enjoying being on clinical placement, they also frequently described a sense of awkwardness which varied between clinical teams and hospital. Various interacting factors – some that acted as facilitators, but more commonly barriers - influenced students’ placement experiences.

Feeling integrated within a clinical team was identified as a key factor for an effective placement, yet students frequently discussed feeling out of place. Lack of integration, feeling inessential and redundant commonly left students describing feeling like a “spare part”, a term cited by multiple participants. Several factors contributed to this during placements.

#### (1) Organisational factors

Students discussed placement structure and the requirements they needed to fulfil during placements. Competing demands such as accessing learning opportunities, attending scheduled sessions and completing workplace-based assessments eroded time available to spend with patients and to integrate meaningfully onto clinical teams. Students contrasted experiences of transience and lack of continuity with exceptional clinical experiences that offered stability e.g., in general practice or emergency departments, where the team was less mobile and more “situated”. Extended periods in one area fostered familiarity and relationships and staff were more likely to take an active interest in students’ learning, involving them in clinical activity. Students valued this as it provided a sense of purpose and allowed application of learning.

*Your timetable works out that you may be on that ward for a couple of days. You’re constantly having to try and introduce yourself to people or ask them what you can do to get involved (P1)*.

*In GP I did feel like we were building trust with them. And that just made the experience so much better. (P17)*

*And people are not willing to teach because they’re like “What’s the point, you’re not here tomorrow. You’re a mere visitor to my workplace” (P5)*.

#### (2) *Student factors*

Students frequently described their inability, through lack of useful skills and inexperience, to effectively contribute to patient care. By contrast to staff and other clinical students e.g., student nurses, medical students generally did not have responsibilities or duties within the clinical team. Their perceived lack of skill and value inhibited them from participating during placements. Their primary role on placement was to learn, therefore benefiting themselves. Some more senior students noted that they had become more assured and validated in their role and were more assertive in the clinical environment.

*I think mainly ‘cause you’re just there to take rather than give before now. I don’t feel like I would have had the right skills to actually give anything useful. (P1)*

*I think because often were in the way, we don’t directly contribute. (P2)*

Some participants also mentioned medical students’ own attitudes and reputations of occasionally being overly confident or displaying a sense of entitlement that did not ingratiate them as newcomers to established teams. These exceptions of behaviour risked leading to medical students generally being perceived poorly.

#### (3) *Environmental factors*

Staff attitudes and behaviours also strongly influenced students’ clinical placement experiences. Teams who weren’t expecting students, were too busy or weren’t interested in engaging with students made placements less rewarding. Students reported experiences of staff, often from other healthcare professions (HCPs), who didn’t engage or occasionally appeared hostile, reinforcing students’ sense of being in the way and making clinical learning additionally difficult to access. These experiences also reinforced professional barriers between professional groups, commonly doctors and nurses. Some commented that although the minority of experiences were negative, these left a lasting impression on them.

The interaction between these various factors effectively produced feedback loops where one barrier or facilitator perpetuates others e.g., once a student identified as being an inconvenience or unwelcome, they were less inclined to ask questions or attempt to get involved in the activity of the ward and vice versa.

*You don’t get to know the staff and they, understandably can get quite annoyed at different medical students everyday coming in, and then having to explain how their ward works every single day - that’s very frustrating so often people’s responses are “Get out of my way. Why are you coming into my team?” (P5)*.

### Early experience of working as a MSHCA

#### (1) *Reasons for undertaking the role*

Participants discussed their inherent desire to support the NHS at a time of unprecedented clinical need; some saw this as being their duty, as future members of the NHS workforce. In this role, supporting service took priority over personal educational goals. Some participants discussed overcoming initial anxiety about being a hindrance at this exceptionally busy time.

Study participants who did not undertake a role as an MSHCA explained various reasons; some returned to alternative roles in the NHS, others needed to personally shield, to care for shielding family members or to provide childcare. Some participants would have appreciated more time and information about roles and contracts but acknowledged the urgency of the situation.

*It was just a way of actually really helping, being in a role where I was actually contributing so I decided to do it (P10)*.

#### (2) *Integration*

Integration with the nursing team was influenced by a number of factors. Where students commenced working with overstretched teams, there were few opportunities for induction. This led to MSHCAs feeling anxious, unsupported and unhelpful. Where clinical colleagues were less welcoming or misinformed about the MSHCA role, integration and acceptance took longer and required proactive efforts by MSHCAs, who regularly had to clarify that they were not in their usual “learner” capacity but would instead be actively contributing, paid members of the team.

Extended placement in a clinical area allowed familiarisation and, from MSHCAs’ perspectives, this signalled their commitment to sharing the workload. By displaying readiness to learn, willingness to undertake tasks, even those not typically completed by medical students, and to take responsibility, students went on to become inducted as effective team-members. Over time, MSHCAs noted better engagement from clinical staff with them.

*I think I had a better time and learnt more, while working because I was actually part of a team, and because I was accepted and, and people put in the effort, both ways…because we were there every day through the thick and thin with them (P5)*.

*One of the nurses was shocked that I was happy to wash a patient by myself, because she was like “No, don’t do that you’re a medical student, you don’t have to do that” and I was saying, “But I do it’s my job and I’m more than happy to”. (P2)*

*I spent a good couple of weeks trying to persuade the HCAs to teach me things (P1)*.

#### (3) *Active participation*

MSHCAs were assigned to various clinical areas including general wards, Covid wards, ITU and discharge planning. Activities undertaken by MSHCAs varied depending on their clinical location. In contrast to experiences of observing or shadowing medical colleagues or being involved in brief clinical encounters, experiences as a MSHCA were active, integrated in care provision and to benefit others. Skills, knowledge and experience acquired allowed participants to be more immediately helpful and to participate in patient care resulting in a sense of satisfaction.

*I felt more capable and independent (P10)*

*It was really nice to be given a sense of responsibility and kind of to be doing, well I was doing the job role* (*P13)*.

*We showed that we weren’t there to follow the doctors around and go to ward rounds, that we were there to make their lives easier (P2)*.

### Learning areas

Although participants were clear that their primary function in the MSHCA role was service provision, as opposed to personal learning, several described insights gained and cited these as being significant formative experiences.

*Sometimes learning, just experiencing and seeing how patients are cared for, how they are washed - I had no idea…(P11)*.

#### (1) *Patient centeredness*

Working as an MSHCA highlighted the extended time that HCAs and other HCPs spend with individual patients, compared to doctors’ relatively brief patient interactions. Medical student patient encounters often focused on single clinical entities and therefore could detract from authentic communication with patients, particularly for novice clinical learners. Close contact allowed carers to gain better understanding of patients’ needs and preferences, greater insights into patients’ wellbeing and the interplay between physical and mental health.

*I also learned how little time you get with patients when you’re a doctor (P10)*

*I think that for me it was the difference in the relationship that we were able to have with patients because it’s completely different to the patient doctor relationship, which is often quite fleeting. You don’t get to really know a person when you’re just seeing them on rounds every day, and that might be it, but seeing them day to day at the most vulnerable and intimate moments as well, I think builds a completely different understanding of a patient’s needs and quality of life…. seeing that, change in their vitals every day or the change in them being able to eat or drink and getting them up and about. I think that’s kind of enriched my medical understanding of how people are going up and down (P1)*.

#### (2) *How the clinical environment and organisation functioned*

By actively participating, students gained more exposure to the healthcare organisation and greater operational knowledge. Useful knowledge and experience gained included understanding the daily ward schedule and seeing how the hospital functioned at evenings and weekends. Previous student experiences had not provided these insights due to their transient and passive observer role.

Y*ou also get a greater appreciation of how a ward works…if one thing goes awry that’s the rest of the day put off. (P5)*

*I learned more about the bleep system, what the structure of a day is. When the physios are around, what the physios do, what the nursing team does in the daytime…. having kind of experienced a snapshot of what it’s like and what working in a hospital is like and how it’s run and the hierarchy … I think that’s going to stand me in good stead (P2)*.

*No one writes about these things anywhere -like there’s a hundred books about getting into medical school, but no one tells you how to help with the bins, and this is how you put the bed down or anything like that (P3)*.

#### (3) *Appreciating other roles; Multi-professional team member*

Students gained understanding of the individual and collective roles of other HCPs. They felt more confident in interacting with other HCP teams and recognised the valuable learning and improved patient care that could be achieved inter-professionally. Some participants commented that doctors appeared to them to have a relatively insular role and may not appreciate the roles of and interface between multiple other teams.

*They know so much about our patients so listening to them… I think that’s just my big takeaway. and that they work so incredibly hard. Seeing all the different roles like the dieticians, OTs, physios I had no idea how often they came along or were involved with patient care. It’s definitely made me appreciate the allied health professionals even more, like the SALT team, physios, OTs - I never appreciated how much they were involved. (P11)*

#### (4) *Affective learning*

For several participants their initial experiences were shadowed with concerns about appropriately using PPE, fear of contracting Covid and concerns about what would be required of them in their role. Participants reconciled some of these apprehensions, noting in future practice they would need to manage these circumstances and responsibilities and therefore this would be useful preparatory experience.

*I’m worried about the future…. about responsibility and… being in situations that I feel out my depth, which is an important thing to get comfortable with when you’re a medical student, but this really forced me to get comfortable, and learn that (P3)*.

Some MSHCAs had their first experience of witnessing a patient die. They were relieved to have experienced this while support from other colleagues was available and prior to future clinical practice.

Students described spending time with patients in their final hours, when families couldn’t visit, and the associated sadness. These were important experiences for developing empathy. They also discussed the joy of witnessing positive patient outcomes after severe illness.

*I’m glad the first time that I’m seeing someone die isn’t when I’m working and it’s my responsibility to you know to actually do something about that….to certify that or whatever. At least now I know what that looks like. (P1)*

### Hierarchies and professional barriers and boundaries

Experiences of medical students being made to feel unwelcome by medics, or more frequently other HCPs, were not uncommon. Medical students observed professional silos as being the norm, noticing that doctors often neglected to consider the opinions of nursing staff. In exceptional clinical areas with “flatter” structures e.g., in emergency department or ITU, team-working and learning was felt by students to be more effective.

Some MSHCAs were immediately welcomed to the teams. Here they received reassurance and support from colleagues and were rapidly involved in activities. Others experienced initial resistance and rejection by clinical staff. Acceptance of MSHCAs by nursing allied professionals required, in some cases, considerable efforts by students in forging relationships and realigning staff expectations of what medical students would be willing to do.

Team working and relationship building helped to transcend some professional barriers. Other experiences unfortunately reinforced traditional barriers such as MSHCAs being ignored by doctors when they were in their HCA uniforms.

*The nurses were great. I think they understood that we were there to learn from them, they could direct us as they pleased because we were there as healthcare assistants. And I think that helped to break down any sort of ego barrier. I have no problem asking a nurse for advice because they know far more than I do. But I think there is still this this perception that medical students think themselves superior to nurses so the nurses, once they realised that wasn’t me, they were really willing to teach us and to help us understand things (P5)*.

*Often doctors will not talk to an HCA, and the HCA is the person who spends the most time with the patient. They know how the patient is doing, often better than the nurses, because they are with them in their most intimate moments, giving personal care, they’ll know how their mental health is probably more so than the nurses…it definitely needs to be team work so that everyone really knows all there is to know about the patient. (P2)*

*I feel like I’m embraced by the doctors and kind of pushed away by the nurses and they kind of don’t want us to be there. But as an HCA I felt like I was embraced by the nurses, but the doctors just didn’t really notice you were in the room (P3)*.

### Influences on professional identity

Participants outlined several areas in which their views had changed during participation and altered their perspectives on what attributes make a good doctor. These resulted in intentions to change their role as a medical student and expressions of how they would like to be as a doctor.

*Because of the experience I’ve had, and how I think it’s changed how I’ll be as the doctor, I think every medical student should have to do it, even if it’s just three months, because it gives you such an appreciation, not only for learning those skills but also for everyone else’s role in the care of patients and how actually, in many cases, if it’s relatively simple management, you might be the least important person in the care as a doctor (P10)*.

*Being an HCA for me will be the best is the best thing about learning medicine, I think you could learn medicine and all the theory behind it but being an HCA for me that’s kind of what taught me how I want to be as a doctor (P14)*.

The importance of working as part of the multidisciplinary team was repeatedly mentioned; with a willingness to participate in patient care when a need arose and an intention to seek and value the opinions of nurses and HCAs who often had closer contact with, and superior knowledge of individual patients’ needs and preferences. This enriched multi-professional perspective may be helpful in promoting humility in medical students, it was noted.

*I would feel far more comfortable talking to a nurse or HCA about a patient, and not just relying on doctors the whole time thinking they’re the ones that will have all the answers because actually the nurses know patients better. And they’ll know who’s a good person to go get an interesting history from, so I definitely feel more comfortable around that and slightly more jaded by doctors! (P10)*

*Seeing the doctors’ role, compared to nurses, definitely influenced how much of a closer relationship I want to have with them in future. And how much I want to utilise their skills what they know. Not just in medical capacity but how much they know the patients as well. (P11)*

Working as part of this team and spending time directly contributing to patient care shaped and informed students’ views on the value of patient-centeredness and holistic care. This had direct effects on reported development of empathy.

*To have empathy, you need to be able to put yourself in their shoes. The best way to do that is if you share the worst times with them and the people who spend the most time with the patients are the HCAs. (P2)*

*Seeing them day to day at the most vulnerable and intimate moments as well, I think builds a completely different understanding of a patient’s needs and on the quality of life that having difficulties in those everyday tasks. (P2)*

Students described the operational insights that they had gained in relation to the clinical environment. These experiences and knowledge would serve them well in their medical training and enhance their confidence for foundation practice.

*I suppose when I present myself it’s more that I’m presenting myself as a member of the team, rather than somebody who has just turned up to be there if it’s okay. (P17)*

*I definitely feel a little bit more empowered to go and find the answers from a clinical perspective as opposed to a textbook perspective.* (*P15)*

Participants started to feel part of “the NHS” and reflected on the positives and challenges of this; students unable to take on clinical roles noted pressures to be involved.

*I feel like the NHS when you’ve worked in it for so long is like a family and I thought, you know, the thoughts of seeing like those people struggling on the front-line*.

*when I knew that I had the skills to help. I felt like I had to step up and do that really, it was something I wanted to do. (P16)*

## Discussion

The overarching concept that emerged from the data was enablement of participation due to increased social capital within the ward Community of Practice [17]. Social capital [18] within the team was earnt by students working alongside staff and demonstrating a willingness to “get stuck in”. This capital then granted students additional opportunities to practice clinical skills and access opportunities to learn from patients and staff. Emergent themes related to barriers and facilitators of participation in the clinical environment, and the developmental outcomes that resulted from participation.

**Fig. 1:**
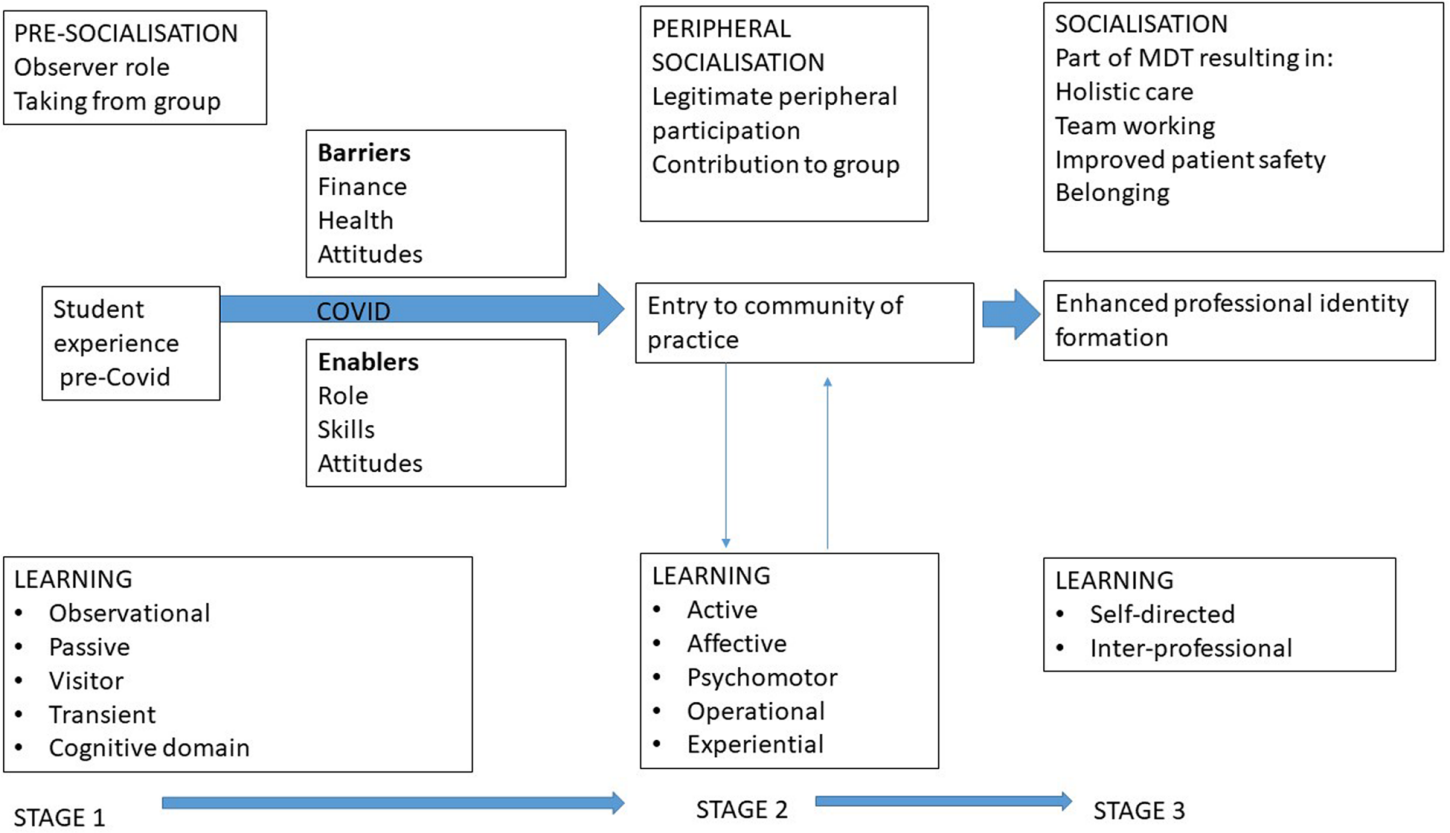
Model describing inter-relation of socialisation and resulting learning from analysis framework

Our findings demonstrate congruence with established social learning & group theories [17, 19–21] and are summarised and demonstrated in Fig. 1.

### A new student role?

Medical student narratives around clinical experiences spoke repeatedly of lack of integration, feelings of redundancy and feeling less entitled to be in the clinical environment, compared to students from other health professions who contribute to care delivery [22]. Changes to healthcare delivery in contemporary organisations [23, 24] have resulted in a shift from a largely apprentice role [25] where students assumed defined roles of “medical clerk” and “surgical dresser”, integrated into a “firm” of doctors and contributing to care, to a largely observational role where students seek discrete “learning opportunities”, “teaching sessions” and “study time”. Though viewed fondly by an older generation of doctors, it must be recognised that this model is not without challenge and does heavily depend on the quality of the “Master” [26].

Efforts have been made to provide students with active roles in different contexts including student-led clinics for underserved populations [27, 28], as Healthcare Assistants [11, 29] and in 3rd sector organisations. These may, however, not translate well to other healthcare systems, lack authenticity and fail to develop a clear medical student role. New possibilities for placements with clear student roles have been highlighted elsewhere [2, 30], we also discovered novel learning opportunities for example NHS 111 and hospital discharge teams.

Recent interest in Entrustable Professional Activities [31, 32] suggests that students can be given incremental medical student roles within the team and be expected to contribute. Students may struggle with self-direction and would need scaffolding within organisational, pedagogic and affective domains [33] to gain progressive independence. This might take the form of a mentor, of a more senior student or other health professional to legitimate junior students to the community of practice [25, 34].

Here MSHCAs received payments for providing service, which may have legitimised the ‘role’ and hence the acceptance into the community. The issue of payment requires consideration if this or a similar model were to be adopted. Integrated, paid placements might have the effect of widening access to medicine, particularly for graduate entrants, many of whom are in financial hardship.

### New models for clinical learning?

The constant expansion of medical knowledge encourages students to approach learning as information acquisition at the expense of clinical learning [35]. The time required to evidence additional extra-curricular activities required of the competitive application process for graduate placements further erodes time for authentic clinical learning opportunities and impacts professional development [36]. These results lead us to question whether medical curricula are in need of further reform to reduce cognitive content and rebalance affective and psychomotor domains of learning [37]. Access to knowledge gets progressively easier through the widespread use of technology and we suggest a shift towards the use of applied information within clinical contexts may be of benefit.

### A new, integrated Community of Practice?

Sharing tasks and commitment to the same goals allowed students to develop relationships, leading to greater acceptance within the team [23]. These interactions with members of the team and closer contacts with patients acted as powerful formative experiences that contextualised learning, demonstrated holistic patient care and the healthcare organisation in action. Integration within a team takes time, and the rapid rotations of medical students inhibit this. Longitudinal integrated clerkships allow students immersion over longer periods of time and may allow deeper integration [38]. Further, a different role, that of HCAs as those described here foreground the development of competencies overlooked in medical placement learning e.g., patient-centred centredness, empathy and multiprofessional identity formation [39]. Specific advantages to integration within a ward-based community of practice included appreciation of the unique skills of these professionals and the considerable contribution this makes to high-quality patient care [40, 41], as well as modelling of values and attitudes that are traditionally more evident in in nursing professionals such as empathy and patient centeredness [42, 43].

### A new professional identity?

Developmental outcomes described by MSHCAs – enhanced empathy, confidence and sense of belonging, greater appreciation of HCP roles and operational knowledge of the clinical organisation, intentions to be a patient-centred future doctor - correspond to those described previously [29, 39, 44, 45] and align with professional values [46].

Doctors have traditionally developed a uni-professional identity [47, 48] and vestiges of a hierarchical structure where doctors sit above other health professionals are still pervasive in some settings and remain part of the “hidden curriculum”. These attitudes and sub-cultures have often acted as a barrier to students accessing the multidisciplinary team community of practice and resulted in difficult relationships with other staff as described by participants.

 Participants observed how poor communication between teams hampered smooth co-working.

Learning within this multi-professional environment has the capacity to reduce barriers to future inter-professional collaboration [48] and consequently, reduce risks to patient safety [49].

In the original study conception, we were interested to explore factors influencing participants’ decision to volunteer as an MSHCA or not. As mentioned, experiences of non MSHCAs are not presented in detail here. While some barriers to participation have been identified, it is worth noting that most of these non-MSHCAs would have been interested in undertaking a role had it been possible and safe for them to do.

## Strengths and limitations

Strengths of the study were the ability to conduct in-depth interviews to saturation. Exploration of typical medical student experience added further strength to our findings of limited integration during placements. Use of framework analysis allowed for robust analysis of the data.

Limitations include that all experiences were gained in secondary and tertiary care, the impact of similar placements in primary care is unknown. Students who self-selected to participate may have been more positive about the MSHCA experience, however non-MSHCA participants widely quoted their peers as having valued the experience. This study was limited to one graduate entry medical school; expansion to other settings would access a more heterogenous sample. Participants’ experiences occurred during the pandemic, a time of global health crisis which undoubtedly inspired altruism and cooperation. Whether similar experiences would be noted by students in typical healthcare delivery conditions *or* whether conditions in healthcare delivery have permanently changed due to the pandemic remains unknown.

## Conclusions

We propose that a longitudinal model of embedment within a single, multidisciplinary healthcare team would benefit & be popular [50] with students, and would add value to the clinical service [51] however, their role and support would need to be considered. The suitability of this model for students with disabilities and caring responsibilities, for whom we are trying to smooth their path into medicine requires careful consideration.

## Supplementary Information

**Additional file 1.**

**Additional file 2.**

## Data Availability

The datasets used and/or analysed during the current study are available from the corresponding author on reasonable request.

## Abbreviations

HCA: Healthcare assistant.
HCP: Healthcare professional.
MSHCA: Medical student healthcare assistant.
NHS: National health service.
OT: Occupational therapist.
PPE: Personal protective equipment.
SALT: Speech and language therapist.
