# Supplementary material for: Qualitative exploration of medical student experiences during the Covid-19 pandemic: implications for medical education
Source: BMC Med Educ. 2021 May 19;21:285. doi: 10.1186/s12909-021-02726-4 (PMC8131173; doi:10.1186/s12909-021-02726-4)
Supplement: Supplementary file 1 — Additional file 1. [file 12909_2021_2726_MOESM1_ESM.docx]

**Appendix 1**

**Participant overview**

| **Participant number** | **Gender**  **F – female, M - male** | **Year of study** | **Role during Covid pandemic** |
| --- | --- | --- | --- |
| P1 | F | 3 | HCA |
| P2 | M | 3 | HCA |
| P3 | F | 3 | HCA |
| P4 | M | 2 | Shielding family member - not HCA |
| P5 | F | 2 | HCA |
| P6 | M | 2 | Providing childcare - not HCA |
| P7 | M | 2 | Shielding – not HCA |
| P8 | F | 3 | Shielding family member – not HCA |
| P9 | F | 3 | Shielding family member – not HCA |
| P10 | F | 2 | HCA |
| P11 | M | 2 | HCA |
| P12 | F | 2 | Shielding family members – not HCA |
| P13 | F | 3 | HCA |
| P14 | M | 3 | HCA |
| P15 | M | 3 | HCA |
| P16 | F | 3 | Returned to previous healthcare professional role |
| P17 | F | 2 | Worked in alternative healthcare service role |
| P18 | F | 3 | Providing childcare – not HCA |
| P19 | M | 3 | Shielding family member - not HCA |
| P20 | M | 3 | HCA |
