# Supplementary material for: Qualitative exploration of medical student experiences during the Covid-19 pandemic: implications for medical education
Source: BMC Med Educ. 2021 May 19;21:285. doi: 10.1186/s12909-021-02726-4 (PMC8131173; doi:10.1186/s12909-021-02726-4)
Supplement: Supplementary file 2 — Additional file 2. [file 12909_2021_2726_MOESM2_ESM.docx]

**Appendix 2**

**Original Interview schedule**

1. Before returning to study graduate entry medicine what was your previous role or occupation?

2. Since the Covid related disruption to your studies what has your role been? What activities have you been involved with?

3. Please tell me about your experiences in this role.

4. How does this role compare to your previous experiences in the clinical environment as a medical student?

5. What has influenced any changes in how see your role as a medical student now?

**Iterative changes**

Following review after the first 4 interviews (two by each researcher), researchers both identified that clear themes were emerging around learning, so a prompt was added if not discussed under question 3;

What did you learn from the experience? Did you learn anything that you would not have learnt routinely during your medical studies?

It was also clear that students were identifying positive aspects of the experience, so a prompt was added to question 5 (if not addressed elsewhere earlier in the interview) to ask if any aspects could be taken forward and integrated into the medical curriculum;

Do you think any aspects from this experience could be taken forward and integrated into the current medical curriculum?
